# Evaluation of Anti-Inflammatory Drug-Conjugated Silicon Quantum Dots: Their Cytotoxicity and Biological Effect

**DOI:** 10.3390/ijms14011323

**Published:** 2013-01-10

**Authors:** Sanshiro Hanada, Kouki Fujioka, Yasuhiro Futamura, Noriyoshi Manabe, Akiyoshi Hoshino, Kenji Yamamoto

**Affiliations:** 1 Vice Director-General’s Lab, Research Institute, National Center for Global Health and Medicine, Tokyo 162-8655, Japan; E-Mails: yfutamura@ri.ncgm.go.jp (Y.F.); nmanabe@ri.ncgm.go.jp (N.M.); ahoshino@ri.ncgm.go.jp (A.H.); backen@ri.ncgm.go.jp (K.Y.); 2 Department of Molecular Cell Biology, Institute of DNA Medicine, Jikei University School of Medicine, Tokyo 105-8461, Japan; E-Mail: kfujioka@jikei.ac.jp

**Keywords:** silicon quantum dot, alminoprofen, cyclooxygenase-2, cytotoxicity and biological effect

## Abstract

Silicon quantum dots (Si-QDs) have great potential for biomedical applications, including their use as biological fluorescent markers and carriers for drug delivery systems. Biologically inert Si-QDs are less toxic than conventional cadmium-based QDs, and can modify the surface of the Si-QD with covalent bond. We synthesized water-soluble alminoprofen-conjugated Si-QDs (Ap-Si). Alminoprofen is a non-steroid anti-inflammatory drug (NSAID) used as an analgesic for rheumatism. Our results showed that the “silicon drug” is less toxic than the control Si-QD and the original drug. These phenomena indicate that the condensed surface integration of ligand/receptor-type drugs might reduce the adverse interaction between the cells and drug molecules. In addition, the medicinal effect of the Si-QDs (*i.e.*, the inhibition of COX-2 enzyme) was maintained compared to that of the original drug. The same drug effect is related to the integration ratio of original drugs, which might control the binding interaction between COX-2 and the silicon drug. We conclude that drug conjugation with biocompatible Si-QDs is a potential method for functional pharmaceutical drug development.

## 1. Introduction

Semiconductor quantum dots (QDs) have the potential to be used in many types of probes and high-efficiency catalysts because of the quantum size effect, which leads to size-tunable band gaps [1,2] and because of their high surface-volume ratio, which enhances the photonic functions and the surface catalytic actions [3]. Over the past two decades, many types of QDs have been synthesized [4,5]. Cadmium-based QDs are of special interest because they have unique photonic properties and photonic stability [6], and they are currently being used in fluorescent biodetection systems [7–9] including bio-imaging performed *in vitro* [10,11] and *in vivo* [12–15]. Our group investigated the efficacy of the QDs modified with various chemicals, antibodies and ligands as specific biological probes [16–19].

Because conventional QDs are made with heavy metal compounds as raw materials, the degradation of such QDs accumulated in the body may lead to cytotoxic disease [20]. Derfus *et al.* [21] reported that cadmium selenide (CdSe) nanoparticles exposed to UV rays released cadmium ions and enhanced cytotoxicity *in vitro*. Mahendra *et al*. [22] also reported the strong alkaline and acid instability of cadmium-based nanoparticles. To prevent these instabilities, commercially available cadmium-based QDs are usually coated with some type(s) of polymers and surfactants. However, an excessive increase in particle size due to the polymer coating of QDs may also lead to a critical accumulation in the body over a long period [23]. Since these negative aspects of the use of QDs for biomedical application have not been resolved, further improved and safer QDs are desired.

As an alternative, carbon group QDs have been developed, including germanium [24–26], silicon [27–30], and carbon (or diamond) QDs [31]. Their quantum yields are low because they have an indirect band gap in bulk properties. However, when the crystal size is minimized to around quantum size, these particles have high-intensity luminescence [32]. In addition, several groups have synthesized silicon (Si) nanoparticles [27–30,33]. Sato *et al*. used a radiofrequency plasma method to synthesize Si-QDs [27], and Warner *et al*. synthesized water-soluble Si-QDs [29] with a liquid phase method. This method can control the surface modification by bonding Si with chemicals that have a C=C double bond. The surface of these Si nanoparticles is protected from their excessive oxidation by oxide passivation or their surface modification.

Nanomaterials have been investigated recently as a carrier for drug delivery systems (DDSs) [34–36]. Liposomal capsules [37] and polymeric carriers [38] such as poly-lactic acid and poly-glycolic acid are already in use as biocompatible materials in gene and drug transfer systems. Since QDs are homogeneously small particles and can be easily modified by surface chemical reactions, QDs that are conjugated by specific biomarkers or medicinal drugs may have potential as DDS carriers. QDs conjugated with medicinal molecules [18] or nucleotides [39] are highly effective for biological applications such as gene transfer and DDSs.

Manabe *et al.* [18] reported that CdSe/zinc sulfide (ZnS) QDs modified with captopril (an antihypertensive agent) enabled the monitoring of a drug’s delivery and performed with almost the same medicinal efficacy as the original captopril both *in vitro* and *in vivo*. Hoshino *et al*. [39] synthesized QDs conjugated with genes such as green fluorescence protein (GFP) and DsRed and performed *in vitro* transfection of the cultured cells. These reports show that the smallness of QDs in itself acquires very specific properties for enhancing their interaction or insertion into cells or biological factors.

In the present study, we synthesized medicinal drug-conjugated Si-QDs for a potential method to be used with functional pharmaceutical drug development. We focused on analgesics (painkillers) because many people around the world suffer from chronic pain and must take painkillers for months, years or indefinitely. There are many types of analgesics, such as paracetamol, various non-steroid anti-inflammatory drugs (NSAIDs), and opioids, all of which have side effects to a greater or lesser extent. For example, possible hepatic impairment is indicated in the drug package insert for paracetamol, gastrointestinal ulcers and allergic reactions are frequent side effects of NSAIDs. Thus, high therapeutic effects and low levels of side effects are strongly desired. Here, we used the NSAID alminoprofen, which is used for rheumatism, as a model drug. We synthesized alminoprofen-conjugated Si-QDs and evaluated the cytotoxicity and medicinal effect of this novel “Si drug”.

## 2. Results and Discussion

### 2.1. Characteristics of Si-QDs

**Figure 1:**
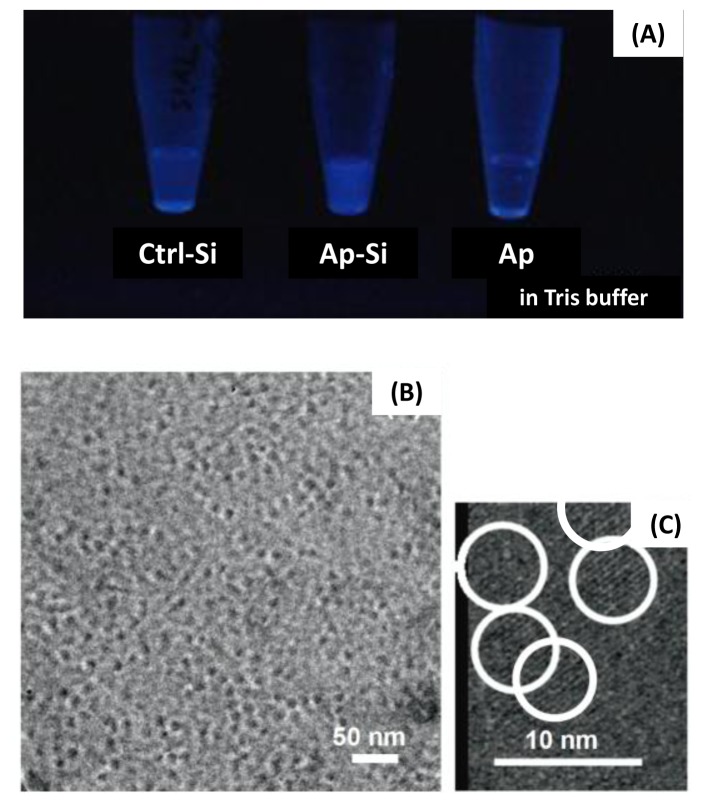
Characteristics of Silicon quantum dots (Si-QDs): (**A**) Fluorescence of each synthesized Si-QDs and alminoprofen under UV-B light. (**B**) TEM image of alminoprofen-conjugated Si-QDs (**B**), and the image at higher magnification (**C**).

We synthesized Si-QDs by the liquid phase method as described by Warner *et al.* [29]. The synthesis of the Si-QDs can bind the surface hydrogen-bonded silicon (Si-H) on the Si-QDs with a variety of chemical compounds with terminal C=C bonds by hydrosilylation using a platinum catalyst, which promotes the formation of a Si-C surface bond. In this study, we modified the surface of the Si-QDs with alminoprofen to form the drug-modified Si-QDs. These hydrophilic QDs exhibit blue photoluminescence in tris-buffer solution (Figure 1A). In the transmission electron microscopy (TEM) images, we observed Si-QDs with diameters of less than 10 nm (Figure 1B,C).

**Figure 2:**
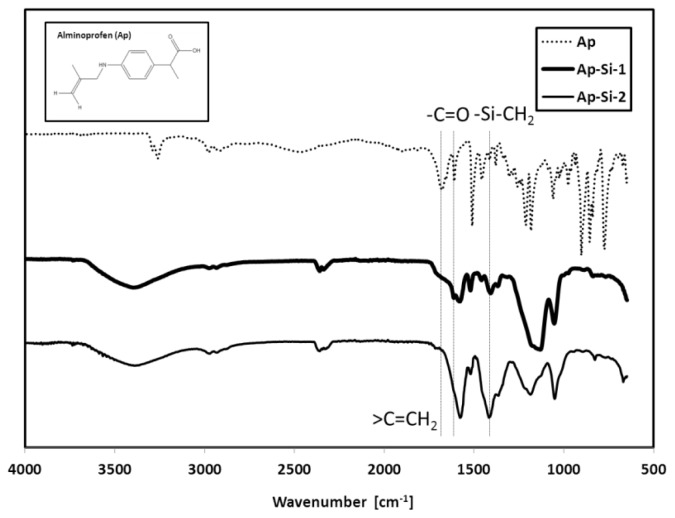
The Fourier transform-infrared spectroscopy (FTIR) spectra of alminoprofen (Ap) and two types of Ap-conjugated Si-QDs (Ap-Si-1 and -2).

**Figure 3:**
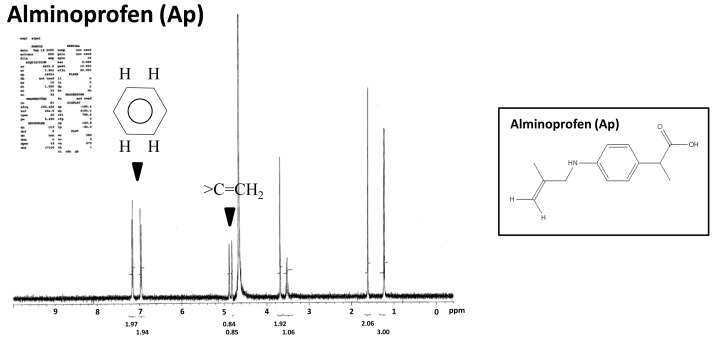

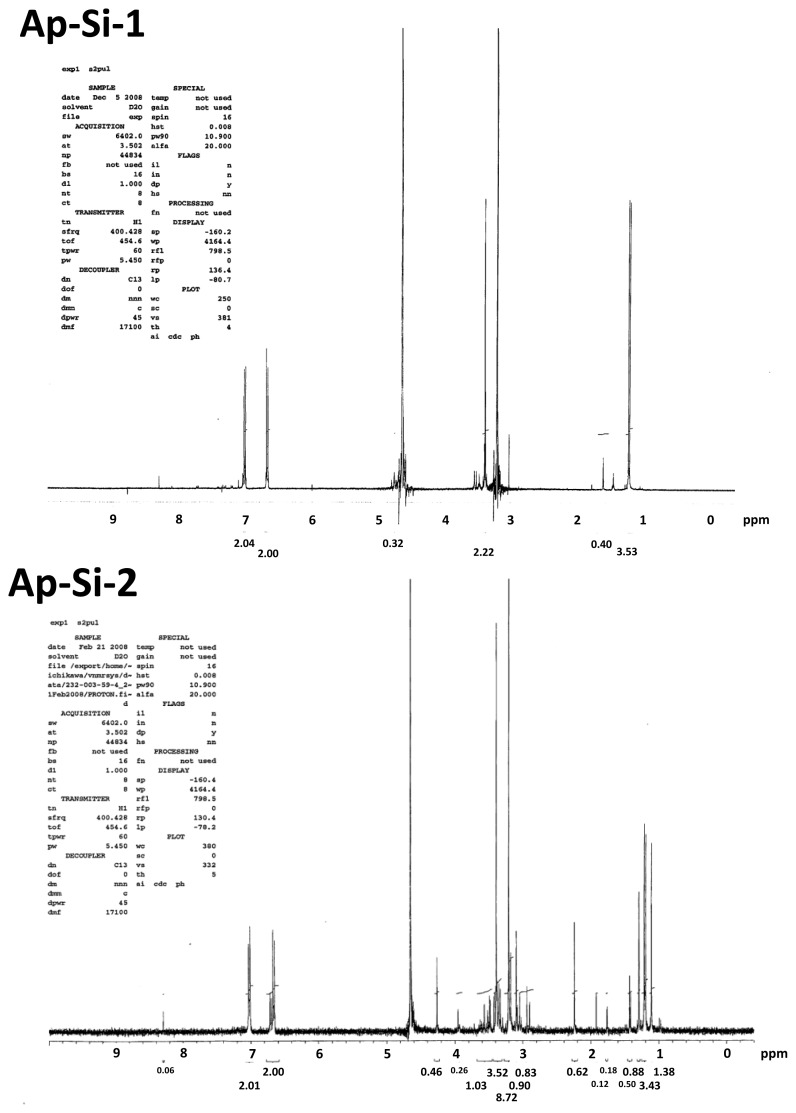
The proton nuclear magnetic resonance (H-NMR) spectra of alminoprofen (Ap) and two types of Ap-conjugated Si-QDs (Ap-Si-1 and -2).

We synthesized two batches of alminoprofen-conjugated Si-QDs (Ap-Si-1 and -2) by slightly different purification processes. To confirm the bonding of alminoprofen to the Si-QDs, we measured the Fourier transform-infrared spectroscopy (FTIR) spectrum (Figure 2) and the proton nuclear magnetic resonance (H-NMR) spectrum (Figure 3). Concerning the results of the FTIR spectrum, we observed the peak at around 1460 cm^−1^, which is thought to be attributable to the vibrational symmetric and scissoring bending of Si-CH_2_ [29], and the results indicate that the drug chemically attached to the Si-QDs. The dominant peak at around 1600 cm^−1^ is attributed to C=O asymmetric stretching vibration of the alminoprofen. The peak at around 1660 cm^−1^ disappeared in the Si-QDs, which indicates that the C=C terminal bond might react with Si-H.

In the H-NMR spectrum, we partially confirmed the formation change of alminoprofen. The alminoprofen peak of C=CH_2_ that ranged from 4.8 to 4.9 ppm disappeared in these two samples, which means that the C=C terminal bonds was opened. An unidentified signal, in part, appeared especially in Ap-Si-2 during the process of the synthesis, but main reaction residues such as tetraoctylammonium bromide (TOAB) were almost completely removed by the purification process.

### 2.2. Cytotoxicity of Synthesized Si-QDs

NSAIDs are inhibitors for cyclooxygenase (COX)-1 and COX-2. The last one is specifically expressed in inflammatory cells, and the inhibition of COX-2 is the mechanism underlying NSAIDs’ ability to attenuate pain. The adverse effects of NSAIDs such as gastrointestinal ulcers and allergic reactions are thought to be caused by the over-inhibition of COX-1 [40].

**Figure 4:**
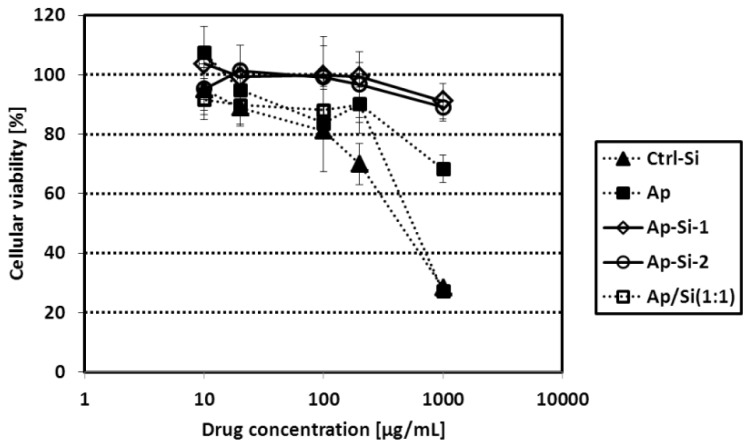
Cytotoxicity of Si-QDs assessed by WST-8 assay for cultured Hep G2 cells. Comparison between Ctrl-Si, alminoprofen (Ap), a mixture of Ap and Si (Ap/Si), and two alminoprofen-conjugated Si-QDs (Ap-Si-1 and -2).

To evaluate the cytotoxicity of Si-QDs, we added the Si-QDs (10–1000 μg/mL) to cell culture using the hepatocarcinoma cell line Hep G2 for 48 h, as described in a previous paper [41], and then evaluated the cytotoxicity by WST assay, which is principally similar to the MTT assay (Figure 4). In the case of bare Si-QDs (Ctrl-Si), we detected significant cytotoxicity at concentrations higher than 200 μg/mL, and we detected slight cytotoxicity in the case of Ap-Si QDs (1000 μg/mL). Alminoprofen, the original medicine, is also detected the cytotoxicity in lower concentration, and the mixture of equal weights of Ctrl-Si and alminoprofen was also significantly toxic compared to the Ap-Si QDs. These results indicate that the drug integration on Si-QDs reduces the cytotoxicity related to the surface interaction between the cells and drug. The reason can be because drug integration reduces both the density of the drug and the drug’s adverse effects per cell.

### 2.3. *In Vitro* Effect of Ap-Si QDs

**Figure 5:**
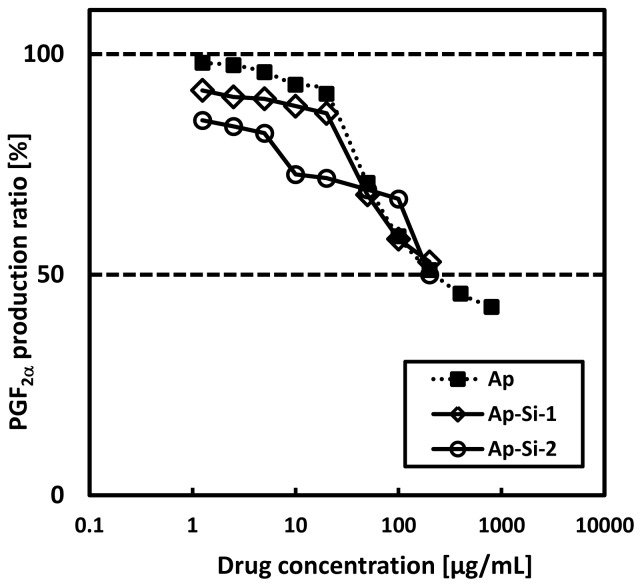
Comparison of inhibition activity of cyclooxygenase-2 (COX-2) using “Si drugs”. Prostaglandin F_2α_ production transformed from arachidonic acid by COX-2 was measured by ELISA.

We evaluated COX-2 inhibition by these “Si drugs” as the biological effect of Si-QDs. We first added each drug to a solution of COX-2, and then arachidonic acid (the precursor) transformed to prostaglandin E_2_ and F_2α_ by COX-2 was added to the solution. After stopping this reaction at 2 min, we measured the prostaglandin F_2α_ by enzyme immunosorbent assay. Compared to original alminoprofen, the Ap-Si QDs revealed the same drug effect per total weight (Figure 5). These results indicate that the Si drugs at least maintain the biological effect and that drug integration on QDs, in some situations, might enhance the biological effect per molecule of the drug.

We can hypothesize that this “Si drug” enhances the interaction of COX-2 by the existence of alminoprofen on Si-QDs or by a non-specific interaction of core Si-QDs that might occur after the first contact of COX-2 with alminoprofen. We calculated the inhibition constant (*K*_i_) as a fitting parameter by Michaelis-Menten analysis. When the *K*_i_ of original alminoprofen was set at 22, the *K*_i_ values of the two Ap-Si QDs were calculated as 0.013, and 0.005, respectively (Table 1), based on their IC_50_ values. These results indicate that enhancement of the interaction between QDs and COX-2 might depend on the number of drug molecules integrated on core Si-QDs.

As we know, in the development of new drugs, it is desirable to: (1) target a specific disease, (2) enhance the medicinal effects, (3) decrease adverse side effects, and (4) maintain, in some case, activity. For targeting specific diseases, some types of organelles, cells, tissues and organs are good candidates. Maeda *et al*. first reported that carcinoma tissues can be targeted using nanosize materials, via the enhanced permeability and retention (EPR) effect [42,43]. This effect depends on the difference of vascular structure between normal tissues and carcinoma tissues, and it leads to a size-selective accumulation of drugs. Yamada *et al.* reported that nanoparticles conjugated with genes or peptides are able to transfer specifically to hepatocytes as a cell-targeting DDS [44]. Thus, drug-conjugated QDs may also have potential as novel drugs, because the smallness of the QDs—which accounts for their high surface ratios—leads to high solubility in buffer and high interactions of various biofactors, and, probably, thus they may be able to target organelles or biomolecules in cells. Hoshino *et al*. confirmed that QDs conjugated with biomolecules can target specific organelles in cells, by a bioimaging analysis [17]. Shiohara *et al.* reported that the toxicity of the conventional cadmium-based QDs depends on the size of QDs or cell types in culture [45], and they noted that the toxicity might be caused by raw materials of the QDs; this would rule out any biomedical applications. In contrast to cadmium-based QDs, the Si-QDs are thought to be biologically inert [41] as mentioned above, unless they accumulate in the body over a long period. Our in vitro results are, so that, promising.

## 3. Experimental Section

### 3.1. Synthesis of Si-QDs

Si-QDs were synthesized by the liquid phase method as described by Warner *et al.* [29]. In brief, SiCl_4_ and tetraoctylammonium bromide (TOAB) were dissolved in toluene and then sonicated. Si-QDs were then formed by the addition of LiAlH_4_ as the reducing agent. The mixture was left to react for 6 h, and then anhydrous acetone was added to stop the reaction.

Surface modification of the Si-QDs was achieved by treating the surface of the Si-QDs with alminoprofen (kindly supplied by UCB Japan, Tokyo, Japan). Alminoprofen-conjugated Si-QDs were formed with H_2_PtCl_6_ in 2-propanol as the catalyst for the reaction between the terminal C=C bond of alminoprofen and the surface Si-hydrogen bonds. After modification of the surface, the sample was purified and filtrated to remove the surfactant and some of the residues [30]. All syntheses were carried out in an argon atmosphere in a glove box to prevent oxidation of the Si (oxygen levels were below 10 ppm).

### 3.2. Measurement of Characteristics of Si-QDs

Confirmation of the Si-QDs was measured by H-NMR and FTIR spectra (Shimadzu, Kyoto, Japan). The particle diameter of the QDs was measured by dynamic light scattering (DLS) using Zetasizer Nano ZS (Malvern, Worcestershire, UK) and measured the QDs’ absorbance with an absorption spectrophotometer (GE Healthcare, Milwaukee, WI, USA).

### 3.3. Cytotoxicity of Drug-Conjugated Si-QDs

The *in vitro* cytotoxicity assay was performed using WST-8 assays. Hep G2 cells were purchased from RIKEN Bio Resource Center (Tsukuba, Japan). They were cultured in DMEM supplemented with 10% fetal bovine serum (FBS), non-essential amino acid solution, HEPES, and antibiotics. The cells were plated at a volume of 1000 cells/well on a 96-well culture plate (Iwaki, Tokyo, Japan) and after 24 h of culture (37 °C and 5% CO_2_) we added various concentrations of solution of Si-QDs. After incubation of the mixtures for 48 h, the cells were washed with Dulbecco’s modified phosphate-buffered saline (D-PBS) twice to remove the nonspecific binding quantum dots.

The WST-8 assay was performed by using the Cell Counting Kit-8 (Dojindo, Mashiki, Japan) with the measurement of the 450 nm absorption of the formazans produced by the mitochondrial activity for 1 h. We calculated the relative cellular viability as the ratio of the absorbance value to that of the QD-untreated cells (as the negative control).

### 3.4. Measurement of Cyclooxygenase-2 (COX-2) Inhibition

We measured COX-2 inhibition for a biological effect by using the COX Inhibitor Screening Assay Kit (Cayman Chemical, Ann Arbor, MI, USA) with the measurement of prostaglandin F_2α_. The enzymatic reaction time was set at 2 min. The evaluated concentration of the drug-conjugated Si-QDs ranged from 1 to 1000 μg/mL by total weight including Si-QDs.

### 3.5. Michaelis-Menten Analysis

The interaction between an enzyme and a drug is described by Michaelis-Menten kinetics, with the following formula:

(1) $$V_{p}=\frac{k_{2}{\left[E\right]}_{t}}{1+K_{m}/[S]+K_{m}[I]/K_{i}[S]}$$

We calculated the inhibition constant *K*_i_. The values *K*_m_ = 2.1 μM, *k*_2_ = 5 s^−1^, [*E*]_t_ = 0.05 μM, and [*S*] = 100 μM were used for each parameters. We estimated the drug/particle weight ratio by the drug concentration measured from 245 nm absorption (Table 1). Each particle weight of QDs was roughly calculated using their DLS size: the average sizes of Si-QDs in buffer solution (0.05mg/mL) were 6.5 nm (Ap-Si-1) and 8.5 nm (Ap-Si-2), relatively.

## 4. Conclusions

We synthesized novel anti-inflammatory drug-conjugated Si-QDs. Compared to the parental alminoprofen, they were less toxic and have the same biological effect. These results suggest that drug conjugation with biocompatible Si-QDs is a potential method for functional pharmaceutical drug development. Our research is a first step toward developing novel “Si drugs” that enhance the functionality of the drug. Further research will be necessary to investigate the underlying mechanisms, with goals such as optimizing the surface modification and controlling the particle size, and expanding to *in vitro* and *in vivo* studies with a dose and time response evaluation.

## Figures and Tables

**Table 1:** The biological effect of two types of Ap-Si-QDs and the original drug, calculated with Michaelis-Menten analysis.

Sample	Particle size (nm)	Weight ratio of Ap	Estimated IC_50_ (mg/mL)	Calculated *K*_i_
Ap-Si-1	6.5	0.23	0.16	0.013
Ap-Si-2	8.5	0.30	0.19	0.005
Ap	N.D.	N.C.	0.23	22

Ap, alminoprofen; Si, silicon; QDs, quantum dots; N.D., not detected; N.C.: not calculated.
